# To S. Strowger

**Published:** 1808-09

**Authors:** 


HARLESTONENSIS.

*August* 10, 1808.

---

### To S. STROWGER.

Sir,

It is not my design to enter into any kind of controversy with you, respecting your observations on a paper, signed Harlestonensis, in the Medical Journal of April; with you, Sir, I can have *no* debate, because I feel it impossible to answer answer what neither I, nor any I have consulted, can comprehend.

You will observe that, in compliance to your suggestion, I have in this instance, assumed the name of

TAYLOR.

*August* 10, 1808.

